# Broadband optical vortex beam generation using flat-surface nanostructured gradient index vortex phase masks

**DOI:** 10.1038/s41598-023-46871-w

**Published:** 2023-11-20

**Authors:** Hue Thi Nguyen, Rafal Kasztelanic, Adam Filipkowski, Dariusz Pysz, Hieu Van Le, Ryszard Stepien, Takashige Omatsu, Wieslaw Krolikowski, Ryszard Buczynski

**Affiliations:** 1 University of Warsaw, Faculty of Physics, 02-093 Warsaw, Poland; 2 Department of Optical Fiber Technology and Quantum Systems, Łukasiewicz Research Network-Institute of Microelectronics & Photonics, 02-668 Warsaw, Poland; 3 Faculty of Natural Sciences, Hong Duc University, 40-157, Thanh Hoa, Vietnam; 4 Molecular Chirality Research Center, Chiba University, 1-33, Chiba, Japan; 5 Department of Quantum Science and Technologies, Australian National University, Canberra, Australia

**Keywords:** Optoelectronic devices and components, Micro-optics, Optical manipulation and tweezers

## Abstract

We developed a new kind of compact flat-surface nanostructured gradient index vortex phase mask, for the effective generation of optical vortex beams in broadband infrared wavelength range. A low-cost nanotechnological material method was employed for this work. The binary structure component consists of 17,557 nano-sized rods made of two lead–bismuth–gallium silicate glasses which were developed in-house. Those small rods are spatially arranged in such a way that, according to effective medium theory, the refractive index of this internal structure is constant in the radial direction and linearly changes following azimuthal angle. Numerical results demonstrated that a nanostructured vortex phase mask with a thickness of 19 μm can convert Gaussian beams into fundamental optical vortices over 290 nm wavelength bandwidth from 1275 to 1565 nm. This has been confirmed in experiments using three diode laser sources operating at 1310, 1550, and 1565 nm. The generation of vortex beams is verified through their uniform doughnut-like intensity distributions, clear astigmatic transformation patterns, and spiral as well as fork-like interferograms. This new flat-surface component can be directly mounted to an optical fiber tip for simplifying vortex generator systems as well as easier manipulation of the generated OVB in three-dimensional space.

## Introduction

Optical beams carrying orbital angular momentum (OAM) which are also known as optical vortex beams (OVBs), are complexly-spatially structured beams with unique properties^1^. An OVB has a central singularity indicating a zero-intensity area surrounded by a doughnut-shaped intensity distribution in its cross section. Each optical vortex beam has an azimuthal phase modulation proportional to a factor of exp(*ilθ*) which is transmitted helically along its propagation axis. The topological charge *l* with integer values shows the order of the vortex beam and associated with it, the amount of OAM carried by each photon equal to *lℏ*^2^. Due to the presence of those unique properties, OVBs have found numerous applications in various research fields^3^. Among others, OVBs greatly contributed to super-resolution microscopy with stimulated emission depletion (STED) technique^4,5^; optical tweezers and spanners^6–9^ in which OAM of the beam imparts a trap and rotation on the particle. OAM-carrying optical vortex beams have been also employed to enhance capacity and security in free-space information transfer^10,11^ and have recently been used to facilitate advances in telecommunications^12,13^. Another application of interest is laser ablation and surface structuring using high-power optical vortex beam as a new materials processing approach to create micro/nano-scaled monocrystalline needles and chiral structures^14,15^. More recently, a new class of coronagraph in astrophysics, so-called stellar coronagraphs, has been developed with the use of optical vortex, to study stellar disks or find planets next to the bright stars^16,17^.

In a broad perspective, optical vortex beams have become important in many scientific and manufacturing applications, thereby there is a demand for efficient and reliable methods of generation of such beams. In general, in order to generate vortex beam with a certain topological charge (certain OAM mode) one must impose a helical structure onto the phase of the incident beam. This can be realized typically in two ways, i.e. using optical phase elements or fiber-based vortex generator^18,19^. In the first method, the implementation of the azimuthal phase term exp(*ilθ*) on the fundamental guided mode is carried out using free-space phase elements such as spiral phase plates (SPPs)^20^, q-plates^21^, computer-generated holograms^22^, and spatial light modulators (SLMs)^23,24^. Among others, SPPs and SLMs are commonly used because the generated beams are stable but they both require bulk setup of discrete optical elements for operation. In the second method, the OAM modes can be generated with the use of specially designed fiber structures like helical fiber Bragg gratings^25^, long-period fiber gratings^26^ and fiber mode selective couplers^27^. Recently, a combination of the standard optical fiber and the SPP element resulting in a compact and robust fiber-based vortex generator has been developed successfully^28^. However, in general, current approaches have certain limitations such as complex optical setup configurations and expensive manufacturing processes, low efficiency, low operating power, large size, low integration ability, narrow operating bandwidths and operation in a particular medium only, i.e. mostly in air.

In the last couple of years, we reported the development of a nanostructured gradient index vortex phase mask (nVPM) for the generation and control of optical vortex beam^29–32^. Those nVPMs were used to convert Gaussian beams into single-charge optical vortices in the visible wavelength regime. The mask was developed employing a low-cost nanotechnological material method which allows creating any refractive index profile for micro-optical components. So far, developed optical components include optical vortex mask^29^, GRIN lenses^33,34^, and axicons^35^. The nVPMs introduce the phase modulation by its internal azimuthal gradient refractive index profile. That means, the vortex beam was generated inside the components so its properties like doughnut-shaped intensity and topological charge value will be preserved in different transparent media including gases and liquids^30^. Moreover, the mask has completely flat-parallel surfaces which can be easily integrated with other optical elements as well as optical fibers. Indeed, we successfully created a robust fiber-based microprobe with an integrated nVPM for fundamental-charge OVB generation^32^. The integrated fiber-nVPM-lens system can effectively generate and, at the same time focus vortices^36^. It should be noted that those nVPMs work well for visible range. However, they show limitations such as very strong light localization in the intensity distribution of the beams or even breaking OAM mode for operating in the infrared range. Besides, those nVPMs are design for specific wavelengths^32^.

In this work, we developed broadband nanostructured gradient index vortex phase masks (BnVPMs) for the generation of optical vortex beams over a wide wavelength range in the infrared regime. The binary structure of the mask consists of 17,557 nano-rods in total. They were made of two lead–bismuth–gallium glasses which are designed and synthesized in-house. To minimize vortex distortion due to waveguiding effect, as we observed in previously fabricated vortex masks^29,30^, we fabricated 19 µm thick mask. Both numerical simulation and experiments verify the feasibility of the conversion of fundamental modes into OAM modes using compact BnVPMs.

## Results and discussion

### Numerical analysis

In order to study the optical beams shaping by the BnVPM, the beam propagation along z-axis through the 19-µm-long component and then in the air was numerically modeled. The dependence of the total phase shift created by the 19-µm-long BnVPMs on the operating wavelength can be determined using the following equation^29^:

1 $$\Delta \varphi =2\pi l\left(\lambda \right)=2\pi \frac{\Delta n\left(\lambda \right){d}_{0}}{\lambda } ,$$

where: Δn(λ) denotes a difference of refractive indices of glasses with minimum and maximum refractive indices forming the gradient index mask for the considered wavelength λ, d_0_—a thickness of the mask, *l(λ)*—a topological charge of BnVPMs for the considered wavelength. As an ideal wavelength independent of vortex mask is impossible to attain, in this work we adopt a pragmatic approach and accept total phase shift is within 10% of the designated $\Delta \mathrm{\varphi }=2\uppi$ value in the near infrared wavelength range to develop spectrally broadband vortex with a constant topological charge *l* = 1. This phase shift corresponds to 10% changes of fractional vortex charge around the nominal integer value (l = 1.0 ± 0.1). The criterion of 10% is somehow arbitrary but reflects our results obtained in the previous study on borosilicate-glass nVPM^32^ where we experimentally realized OVBs with the same quality and characteristics even though the topological charge was within 10% of its nominal value.

**Figure 1:**
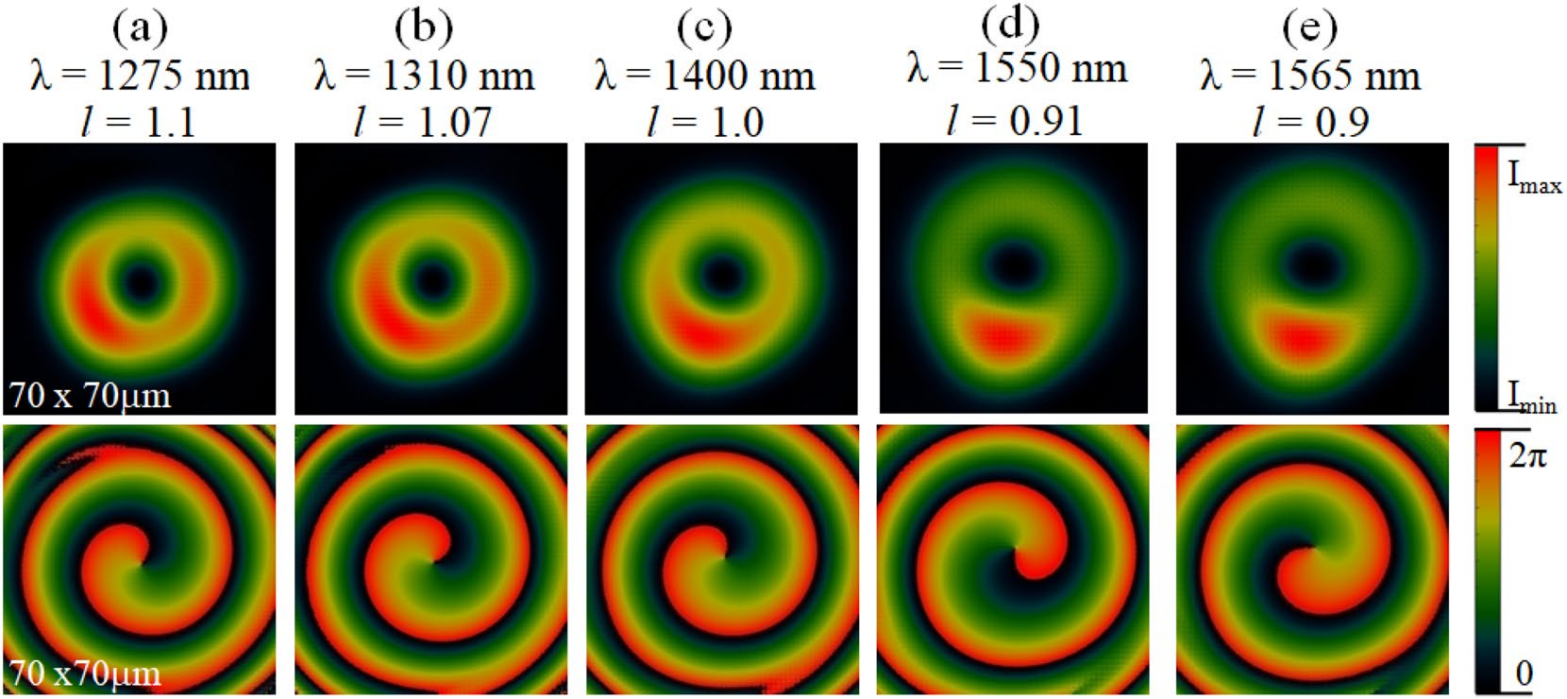
Numerical simulations of the optical performance of the designed BnVPM in the generation of OVBs for the wavelength range from 1275 to 1565 nm: the cross-sectional intensity profile (top row) and phase structures (bottom row). The numerical results are visualized at a distance of 500 µm from the output end of the mask. The *l* denotes the topological charge of the generated vortex beam.

For all simulation tasks, we used in-house developed software employing the Fourier transform beam propagation method (BPM)^37^. It is assumed that all the computational simulations in this work are conducted with the use of a planar beam and with Gaussian intensity distribution as the input source. The incident beam is limited by a circular aperture with the diameter of 25 µm. A few input wavelengths were considered, namely (λ = 1275; 1310; 1400; 1550; 1565 nm). The computational window has the size of (*x,y,z*) = 200 × 200 × 500 µm. The lateral resolution is fixed at 0.1 µm. The longitudinal resolution varied between 0.05 and 0.5 µm, for the propagation inside the mask and in air, respectively. The wavelength dependence of calculated output intensity and phase distribution at a distance of 500 µm from the output end of the mask is shown in Fig. 1.

The results confirm that masks generates a high-quality optical vortex beam with topological charge *l* = 1 at 1400 nm wavelength depicted in Fig. 1c. The beam has a relatively uniform doughnut-like intensity distribution due to the reduced influence of the waveguide effect when the light beam propagates in 19 µm thin medium. It also shows a correct single-twist helical structure with the phase singularity located exactly at its center.

In addition, it can be seen that the fundamental-charged OVBs with the fractional topological charge varying within the range of 10% (l = 0.9–1.1) are obtained at all other considered wavelengths from 1275 to 1565 nm (Fig. 1a,b,d,e). All these results show the corrected phase structure with single arms and doughnut-like intensity profiles. These numerical results confirm that the designed BnVPM works well for the whole wavelength range of 290 nm from 1275 to 1565 nm. Notice that the quality of the vortex beam generated with a given mask is significantly better for shorter wavelengths. This is very likely caused by the excess of the winding phase which exceeds 2π for shorter wavelengths. For longer wavelengths, the phase singularity is, in fact, incomplete so the beam experiences stronger deformation in propagation with visible waveguiding effect.

## Experiment

**Figure 2:**
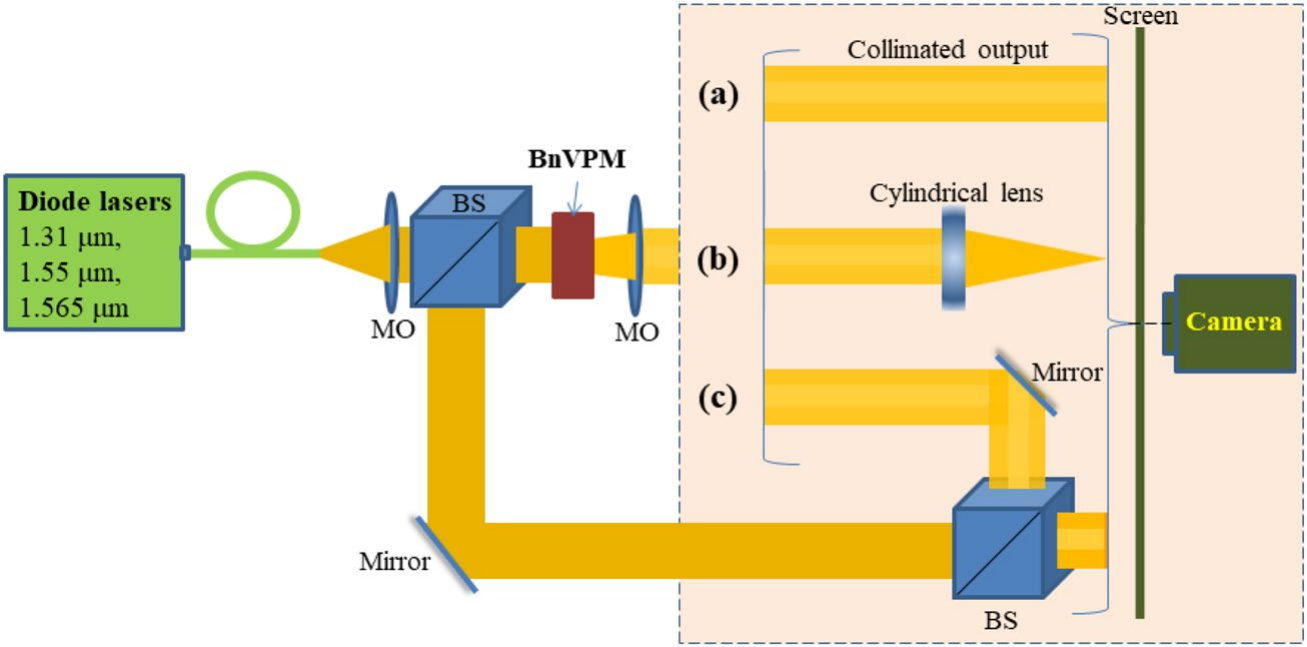
Schematic of measurement setups for studies on the ability of the fabricated BnVPM on the generation of optical vortex beams in a broadband infrared wavelength range. Setups for observing doughnut-like intensity distribution (**a**); for observing the phase singularity and determining the topological charge of the generated OVBs using astigmatic transformation (**b**); and Mach–Zehnder interferometer configuration (**c**).

This section is devoted to an experimental verification of the optical performance of the fabricated BnVPM to generate of OVBs at several wavelengths mentioned above (*λ* = 1310; 1550; and 1565 nm). We note that optimum performance with vortex topological charge *l* = 1 is expected for the wavelength of 1.4 µm. However, due to a lack of an appropriate laser source we carried on experimental work for a few wavelengths at both sides of the optimum wavelength. For that purpose, we used three pigtailed single mode diode laser sources (Butterfly-laser-diodes, Thorlabs). In the measurement setup, our fabricated BnVPM sample with a thickness of 19 µm was mounted to the in-house 3D-printed holder. The whole system was attached to a 3-axis stage (Nanomax, Thorlabs) for precise control of the sample position in space. All measurements were carried out using the same optical setup, with only laser sources being changed between measurements. The incident beam from the light source was collimated by a microscope objective (MO, magnification ×20 and NA = 0.35) before illuminating the BnVPM sample. It should be noted that the illuminating beam size should correspond to the aperture of the BnVPM to ensure the effective generation of vortex beams. The output beam that emerged from the sample was collimated by another MO (magnification ×20 and NA = 0.35). Then we used three techniques (Fig. 2) to verify the characteristics of the generated OVBs namely, doughnut-like intensity distribution, phase singularity and topological charge.

The first setup (Fig. 2a) is used to record the cross-sectional intensity pattern of the beam. Herein, the collimated output beam generated by the BnVPM was projected on a screen and recorded by IR camera. The distance between the output end of the BnVPM and the imaging system (screen and camera) was fixed at 50 cm. The second setup (Fig. 2b) was used to study the effect of the astigmatic transformation on the generated vortex beam^38^. It allows for a quick determination of the topological charge of the beam. The main part of the first experimental setup was kept unchanged for the generation of OVBs, but a cylindrical lens was placed in front of the screen at a distance exactly equal to its focal length of *f* = 7.5 cm to observe the characteristic pattern of bright and dark stripes on the screen.

Another common analysis of phase singularity and topological charge of an OVB involves observation of interference patterns resulted from the interference between OVB and a reference Gaussian beam. This was realized in Mach–Zehnder interferometer configuration^39^ as shown in Fig. 2c. A fork-like or spiral interference patterns were formed when the generated vortex beam and the Gaussian reference beam interfered non-collinearly and collinearly, respectively. The appearance of additional fringes coming out from the central zero-intensity region in the fork-like structure confirms the phase singularity as well as indicates the topological charge value of the generated OV beam. This would be also indicated by the number of spiral arms emerging from the center of the co-axial interferogram.

**Figure 3:**
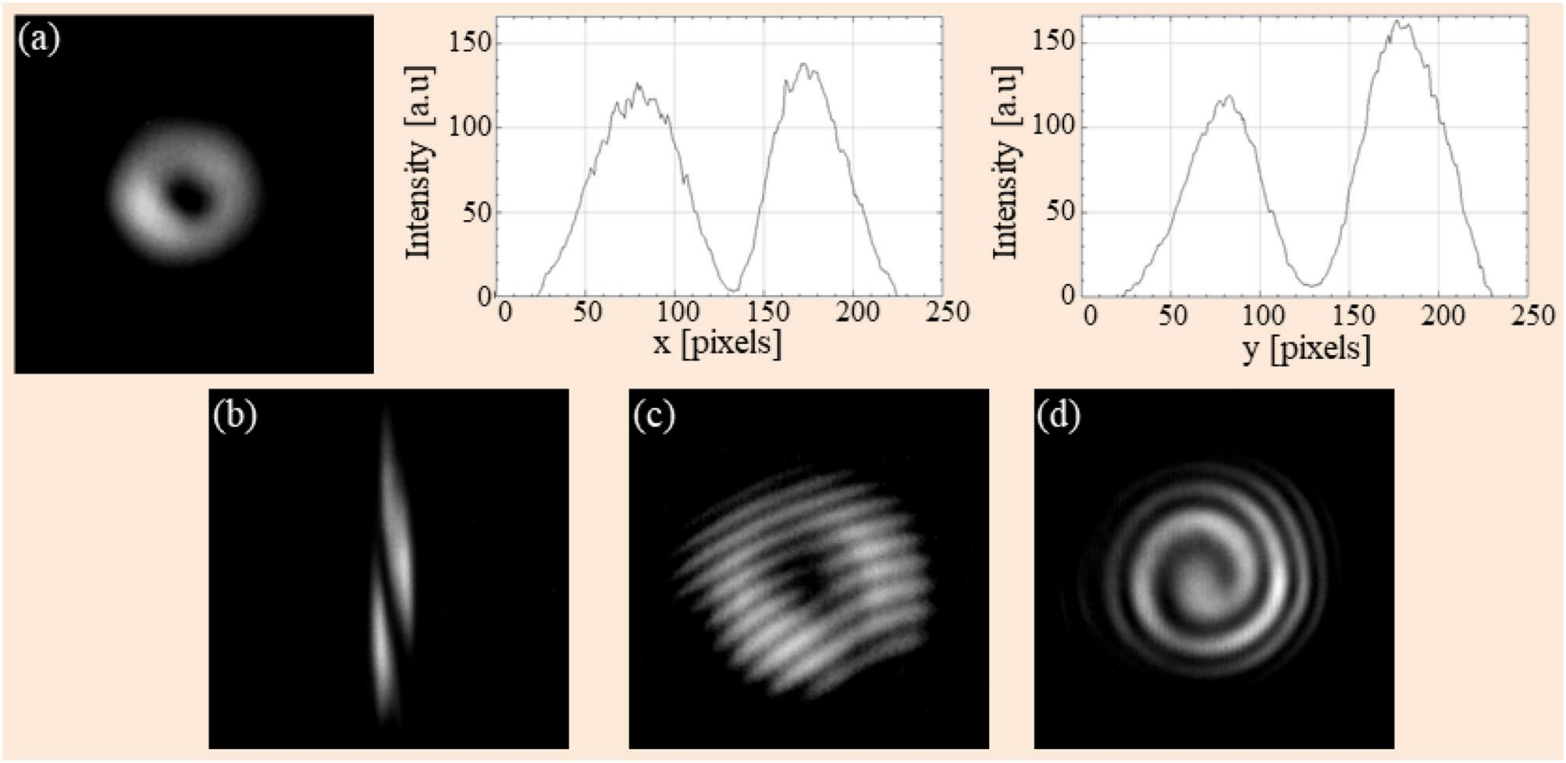
Experimental results for verifying the single-charge OVB generation at λ = 1310 nm: doughnut-like intensity distribution and its horizontal and vertical profile (**a**); astigmatic transformation pattern of the generated vortex (**b**); fork-like (**c**) and spiral (**d**) interferograms realized in Mach–Zehnder interferometric configuration.

The experimental results at the wavelength λ = 1310 nm are shown in Fig. 3. Figure 3a depicts the experimentally observed annular character in the output beam’s intensity distribution. Its clearly dark central region indicates the existence of phase singularity of the beam. This can be also seen in the graphs in Fig. 3a describing horizontal and vertical intensity profiles of the vortex cross-sectional pattern. The intensity distribution on the ring-shape is relatively uniform, showing a weak influence of the waveguiding effect when the light propagates inside such a thin nanostructured component (thickness of 19 µm). Figure 3b presents the results of the astigmatic transformation of the generated beam using the setup in Fig. 2b. We clearly see that in the astigmatic intensity pattern consists of one elongated dark region clearly separating two bright stripes, indicating the single topological charge* l* = 1 of the output beam.

Those results were further confirmed by analysis of the interference patterns observed in the setup as shown in Fig. 2c. We obtained the fork-like (Fig. 3c) and spiral (Fig. 3d) pattern corresponding to non-collinearly and collinearly interference between the vortex beam and Gaussian beam, respectively. In the fork-like structure, there is one extra fringe coming out from the central zero intensity region substantiating the axial phase singularity as well as a single topological charge of the OVB. Moreover, this is further confirmed by the spiral pattern—a single spiral arm surrounding a singularity point at its center. All these results demonstrate that our BnVPM can experimentally convert a Gaussian beam into an optical vortex beam with topological charge* l* = 1 at the wavelength of λ = 1310 nm.

**Figure 4:**
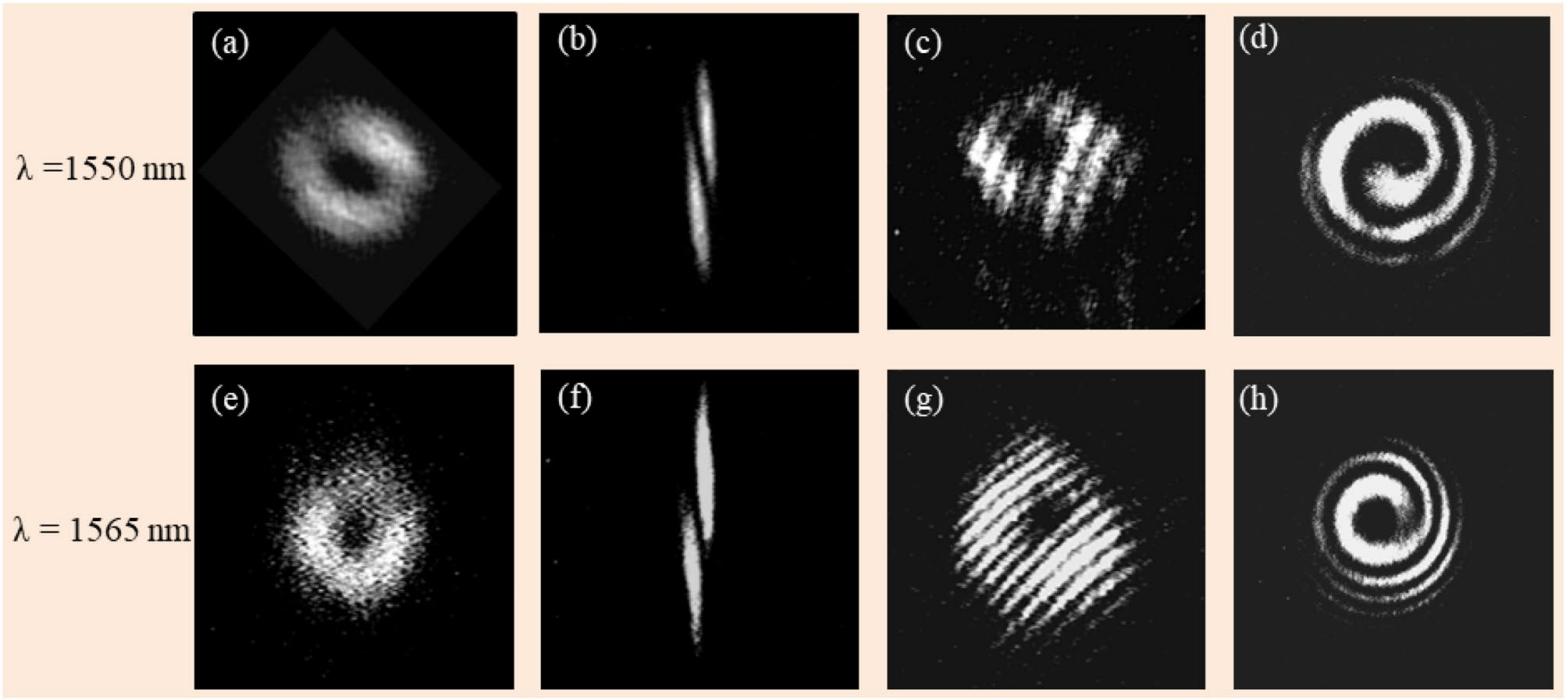
Experimental results for verifying the single-charge OVB generation at λ = 1550 nm and 1565 nm: doughnut-like intensity distribution (**a**,**e**); astigmatic transformation pattern of the generated vortex (**b**,**f**); fork-like (**c**,**g**) and spiral (**d**,**h**) interferograms realized in Mach–Zehnder interferometric configuration.

In the next set of experiments we used BnVPM at the two longer wavelengths λ = 1550 nm and 1565 nm. The results are shown in Fig. 4.

It can be seen that BnVPM performed similarly to the case λ = 1310 nm case, demonstrating its ability to efficiently generate optical vortex beams with topological charge* l* = 1, as there are doughnut-like intensity patterns with the visible zero-intensity central regions. The single charge value *l* = 1 of the vortices is ascertained by the presence of a single dark elongated strip in the astigmatic transformation pattern and the presence of only one extra fringe, and single spiral arm in the corresponding interferograms. Those experimental results are agree with our numerical simulations. Notice that the quality of the vortex beams generated in these experiments deteriorates as the wavelength departs from the optimal value of 1310 nm. As elaborated earlier, this effect is a result of the incompleteness of the winding phase, so the beam experiences diffraction and waveguiding, resulting in stronger nonuniformity.

## Discussion

We have developed new nanostructured gradient index vortex phase masks for the generation of optical vortex beams in a broadband infrared wavelength range. The vortex component was designed and fabricated using the nano structurization technique as described in^29^. It is composed of nano-rods made of two kinds of in-house developed lead–bismuth–gallium glasses which are characterized by wide transmission window up to 4.5 μm. This pair of glasses have a high refractive index difference (∆*n* = 0.073 ÷ 0.708 for near infrared wavelength range up to 1.7 μm), that requires thin BnVPMs (*d* ≤ 23 μm) to create the full 2π phase modulation.

As a demonstration, the BnVPM was designed and fabricated with 17,557 nano-rods in the entire structure. It had a diameter of 29 μm and a thickness of 19 μm. We experimentally demonstrated that the mask allowed the conversion of Gaussian beams into a high-quality vortex beam with topological charge *l* = 1 at three wavelengths λ = 1310; 1550; and 1565 nm. In addition, the numerical simulations showed that the investigated BnVPM should work well for a total bandwidth of ~ 290 nm from 1275 to 1565 nm.

The fabricated BnVPMs have completely parallel surfaces which allow simple integration with other components and optical fiber to form a compact fiber-vortex component optical system offering new functionalities. Similarly to the previously considered by us vortex phase masks, our new vortex converter introduces the angular phase shift via the internal refractive index profile. Therefore, their performance does not depend on the surrounding transparent media. This means that developed flat-surface nanostructured vortex phase masks that work in a broadband infrared range and are fiber-based, are beneficial for various applications in infrared regime, such as particle manipulation and material processing.

Importantly, those results confirmed the flexibility of our proposed fabrication method which allows designing VPMs to work for different wavelength ranges depending on the available glasses. This is promising for the generation of optical vortex beams with unique properties like achromatic vortices, and white vortices. It is should also note that the used fabrication method is cost effective and can be used in mass-manufacturing of optical elements. From one initial preform, we obtain tens of meters of sub preform with different diameters after the first thermal process. Those can be used furher to fabricate thousands of BnVPMs or BnVPM array elements. Moreover, from one structured fiber, we can fabricate BnVPMs with different thickness for generation of OVBs with different topological charges at different wavelengths.

## Materials and methods

### Glasses selection and design of nanostructured gradient index vortex phase masks

In principle, a gradient index vortex phase mask modulates the phase of the incident beam by continuous azimuthal variation of its refractive index profile. In particular, the refractive index of the cross section of the mask is constant in the radial direction and changes linearly with azimuthal angle *θ: n(θ)* = *n*_*0*_ + *θ(*n_1_ − n_0_*)/2π*, where n_1_, n_0_ is the highest and the lowest refractive index value of the material of the mask. Due to the current technological limitations, the realization of azimuthal refractive index profile in a continuous solid medium is a big challenge, especially at microscale. In 2017, we employed the binary nanostructuring technique to successfully create, the vortex phase micro-optical components (nVPM)^29^. By using this method, we designed nanostructured masks that consist of thousands of parallel nano-sized rods. Each of those rods is made of one of two types of glasses, with different refractive indices corresponding to the highest and the lowest values n_1_(λ) and n_0_(λ), respectively. The effective refractive index profile introduced by the binary-nanostructure component is defined by the arrangement of those nano-sized rods constituting the structure of the nVPM. This approach is based on effective medium theory (EMT) which is described by the Maxwell–Garnett effective medium approximation model^40^.

However, the previously fabricated nVPMs worked well only for wavelengths shorter than 1 μm^30,31^. The reason for that is that the nVPM with a constant thickness d_0_ can create an angular phase modulation at a certain input wavelength λ equal to Δφ = 2πΔ*nd*_*0*_/λ with Δ*n* = *n*_1_ − *n*_0_. To generate an optical vortex beam with topological charge* l*, the required phase modulation of the beam depends on two factors, vortex mask length and refractive index difference between low and high refractive index areas can be calculated with Eq. (1).

To generate optical vortex beams at longer wavelengths, the nVPMs should be thicker than the ones used for the shorter wavelengths. However, the thicker mask suffers from, the so-called, waveguiding effect in which the light tends to localize in the area of high refractive index. Consequently, this leads to nonuniform intensity distribution in the beam cross-section or even to the broken doughnut-shape of the OV beam^31^. Therefore, for designing an optical vortex mask working in infrared wavelengths which should be thin enough to reduce the light localization, the most important parameter needed to be controlled is the refractive index difference of glasses used. This means that by using the same process and only changing glasses to ones with different dispersive properties we can design different nanostructured vortex phase masks. By choosing either commercially available pairs of glasses or synthesizing our own we can create nVPMs with different working wavelength ranges.

In this work, we aimed to design a broadband nanostructured vortex mask (BnVPM) working in near infrared regime. To achieve this, the first condition is that the pair of glasses should have the broadband transmission in near infrared range. Secondly, their thermal and mechanical coefficients need to be matched for multiple thermal processes. Thirdly, the refractive index difference must large enough for nVPM to be sufficiently thin, but be still greater than 15 μm which stems from the fabrication constraints. Finally, and most importantly, the broadband property means the BnVPM should have low sensitivity to wavelength in a given range. Formally, this means that the variation of phase for the wavelength:

2 $$\frac{\Delta \varphi }{\partial \lambda }=2\pi l{d}_{0}=2\pi \frac{1}{\lambda }\left(\frac{1}{\lambda }-\frac{\partial \Delta n}{\partial \lambda } \right)$$

should be as close to zero as possible. In principle, this requires finding materials whose dispersion would behave as 1/λ. Since this is rather impossible, one can minimize Eq. (2) by making the vortex mask as thin as possible. Considering that *d*_*0*_ = *lλ/*Δ*n*, the last requirement means that we should aim at materials with very high refractive index contrast.

Based on those conditions, we developed a pair of lead–bismuth-gallium glasses labeled CS740 and CS1030^41^. The glasses were in-house synthesized by the melt-quenching approach at Łukasiewicz Research Network-Institute of Microelectronics & Photonics (IMiF). The glasses are composed of commercially available components SiO_2_, B_2_O_3_, Ga_2_O_3_, PbO, and CdO whose molar mass percentages are given in Table 1.

**Table 1:** Chemical composition of the lead–bismuth-gallium glasses.

Glass label	SiO_2_	Bi_2_O_3_	Ga_2_O_3_	PbO	CdO
CS740	40%	10%	13%	30%	7%
CS1030	30%	14%	16%	30%	10%

**Figure 5:**
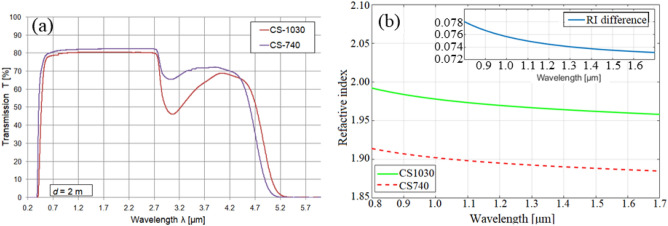
(**a**) Transmission properties of CS1030 and CS740 glasses measured in samples of 2 mm thickness; (**b**) wavelength dependence of refractive indices of the CS glasses and their refractive index (RI) difference.

The developed glasses are 5-compound lead–bismuth–gallium systems that are characterized by broad transmittance windows in the infrared wavelength range, making those glasses having a good transparency from visible up to 4.5 μm as shown in Fig. 5a. It can be also seen that the transmittance range is shifted to a shorter wavelength direction for the glass with a higher content of SiO_2_.

Importantly, the presence of the two additional oxides of SiO_2_ and CdO can reduce the tendency of the lead–bismuth–gallium systems to crystallize^41,42^. As a result, the crystallization resistance and thermal stability of both optimized glasses are significantly enhanced, which makes them highly compatible materials with multistage thermal processing. Notably, those glasses provide good rheological properties and relevant expansion coefficients as well as thermal matched coefficients^37^. The viscosity-temperature characteristics measured by the dilatometric setup and in the heat Leitz microscope showed very similar values for CS1030 and CS740. The difference in curvature temperatures and in sphere temperatures between those glasses are relatively low, ΔT_c_ = 50 °C and ΔT_sph_ = 60 °C, respectively. Simultaneously, the coefficients of thermal expansion are similar for both glasses and is α_CS1030_ = 83.8 × 10^−7^ K^−1^ and equal to α_CS740_ = 81.3 × 10^−7^ K^−1^ in the range from 20 to 300 °C. The difference α_CS1030_ = 83.8 × 10^−7^ K^−1^ and equal to α_CS740_ = 81.3 × 10^−7^ K^−1^ reported in^37^. Those allow the two soft glasses to be thermally processed together and ensure limited stress during cooling. Reduction of cooling induced stresses is especially important as it may lead to fabricated structure cracking and warping.

The dispersion properties of the developed CS glasses were measured using a Michaelson interferometer. The measurement range was in 0.5–1.7 μm range. Based on those experimental results, the Sellmeier coefficients (B_i_ and C_i_) were determined as in Table 2 following Sellmeier’s formula:

**Table 2:** Sellmeier coefficients for the lead–bismuth-gallium glasses.

Sellmeier coefficients	CS1030	CS740
B_1_	2.15976	2.42275
B_2_	0.22578	0.135667
B_3_	1.97162	1.50
C_1_ [μm^2^]	0.02412	0.02261
C_2_ [μm^2^]	0.09301	0.09297
C_3_ [μm^2^]	195.0	153.49733

3 $${n}^{2}\left(\lambda \right)=1+ \sum_{i=1}^{3}\frac{{B}_{i}{\lambda }^{2}}{{\lambda }^{2}-{C}_{i}}$$

These optimized glasses CS740 and CS1030 are characterized by very high refractive indices i.e. *n*_CS1030_ = 1.9665 and *n*_CS740_ = 1.8925 measured at wavelength λ = 1310 nm. The increase in the concentration of the low molecular weight component SiO_2_ in glass composition results in a decrease of the refractive index for CS740 glass. CS1030 has a higher refractive index for the whole considered wavelength range.

Figure 5b presents the wavelength dependence of the refractive index of the CS glasses calculated using the Sellmeier coefficients obtained in Table 2. Their refractive index difference is relatively high Δ*n* = 0.073 ÷ 0.078 for near-infrared considered wavelength range) as shown in the sub-figure. These are about three times higher than those for the NC-glass nVPM (Δn_NC_ ≈ 0.025) at the same wavelength reported in^30^. As a result, the thickness of CS-glass BnVPM required for a 2π phase shift modulation in order to generate OVBs with topological charge *l* = 1 is about three times smaller than for NC-glass nVPM at the same considered wavelength^30^.

In the nanostructuring approach, the binary structure of the BnVPM was designed and optimized based on effective medium theory^40^ and simulated annealing approximation with an in-house developed algorithm^43^. In this approach, using the refractive index profile of the developed CS glasses, we first determined the target continuous refractive index 2D map of the cross-section of the vortex component as mentioned above. Then, we designed its corresponding binary structure composed of spatially distributed rods made of CS1030 and CS740 glasses. In particular, the effective permittivity (consequently effective refractive index) for all nano-rods of the binary dielectric-material structure is assumed to be a weighted average of the effective permittivity of the particular rod and of its neighborhood nano-rods according to the effective medium theory. The distribution of those nano-rods is optimized using simulated annealing approximation^43^ in such a way that the final structure will provide an effective refractive index profile as close as possible to the desired continuous gradient refractive index target. The details of the design and optimization process of the binary nanostructure pattern of a BnVPM were discussed in our previous works^29,30^. It should be noted that the condition for a binary medium to be able to be considered as an effective continuous material is the size of the individual rods in the structure. The diameter of those rods must be smaller than the operating wavelength. Otherwise, the diffraction effects of individual elements become considerable and degrade the component’s performance^44^. The reported threshold value was λ/2π^40^, but recently, this value was reduced to λ/3 by controlling the diffusion during the drawing process^45^.

**Figure 6:**
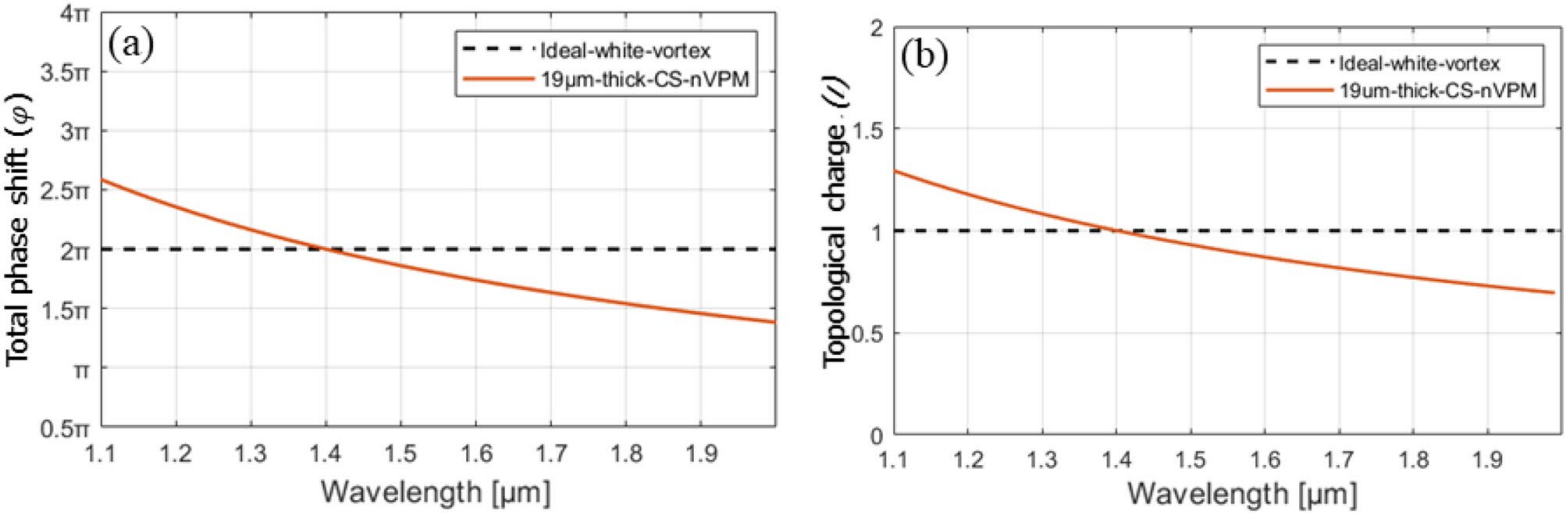
The phase shift in the nanostructured vortex phase mask composed of two CS1030 and CS740 glasses with a thickness of 19 µm (**a**) and the topological charge of generated vortex beam (**b**) for various wavelengths.

To model the broadband performance of the vortex mask we used Eq. (1). The optimum thickness *d* of BnVPM with topological charge 1 can be derived from Eq. (1) in the form $d=\frac{1}{n(\lambda )}\lambda$, where λ denotes the wavelength in the center of the considered infrared range. For the selected wavelength of 1400 nm the optimum thickness of BnVPM is 19 µm for the considered pair of glasses and corresponds to 2π phase shift in the mask. With Eq. (1) we can further calculate the dependence of the phase shift in the mask for other wavelengths (Fig. 6a). The obtained phase shift characteristics is very flat due to the specific relation between material dispersion as shown in Fig. 5b. Similarly the topological charge of generated OVB can be calculated for the range of wavelengths with Eq. (1). A slope of this characteristic is also very low and proves small changes of the topological charge with wavelengths (Fig. 6b).

**Figure 7:**
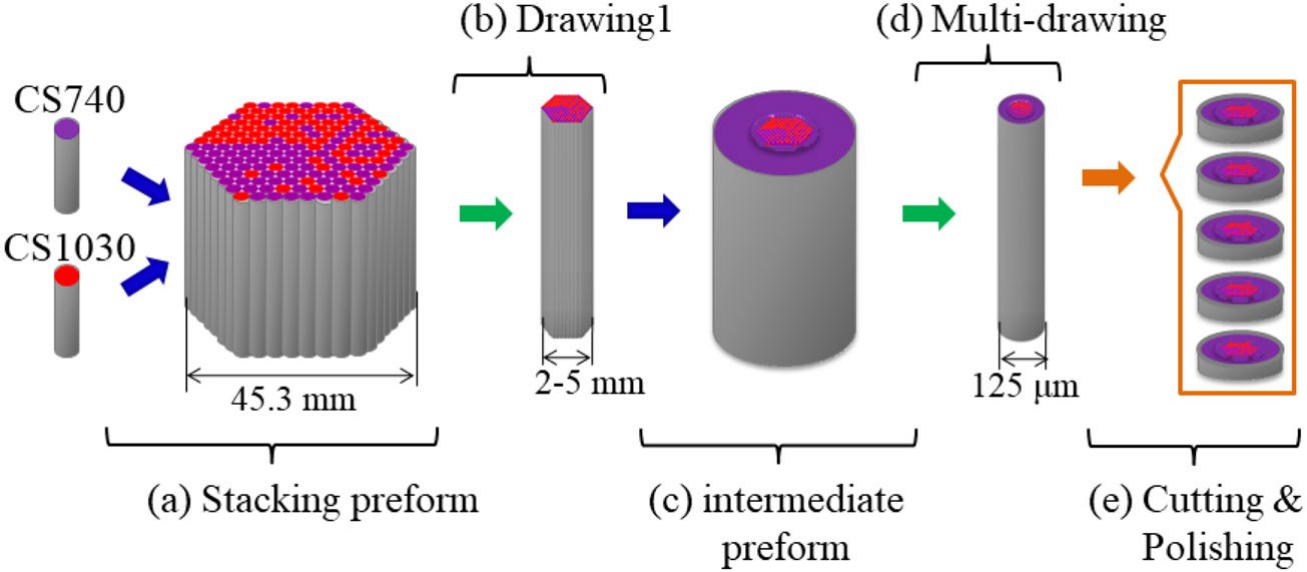
Schematic of fabrication procedure of the BnVPM components using CS740 and CS1030 glass rods.

Our binary nanostructure BnVPM was fabricated using the stack and draw technique. This method is commonly applied for the development of photonic crystal fibers but can be easily modified for the fabrication of BnVPMs^29,30^. The schematic of the fabrication procedure is represented in Fig. 7. The procedure begins with manually stacking a preform (following the designed pattern) using two kinds of CS-glass rods with a diameter of 0.3 mm and a length of 11 cm (Fig. 7a). This initial preform has a diameter of 4.53 cm. Then, in the first thermal step, the preform is drawn down to a diameter of around 2–5 mm in the fiber drawing tower, as shown in Fig. 7b. The temperature of the glasses during the thermal process has to be kept in range between curvature and sphere creation to maintain proper viscosity for drawing^37,44^. In the next step, this structure is placed inside a glass capillary made from lower-RI glass CS740 (Fig. 7c), making an intermediate preform. In the second thermal step (Fig. 7d), we do multiple drawings of the intermediate preform. The multi-drawing process finished when the diameter of the drawn structure reached the standard size of the optical fiber (125 μm). In the last step of the fabrication procedure (Fig. 7e), the final drawn structured fiber is cut and polished until the designed thickness is obtained. This nanostructuring technique offers high control and reproducibility and is ready for mass fabrication. From one stacked preform, we can obtain thousands of identical-structured elements with thicknesses of tens of micrometers. In addition, it is possible to obtain vortex elements with different diameters in a single process by adjusting drawing parameters like temperature, feeding, and drawing speed.

**Figure 8:**
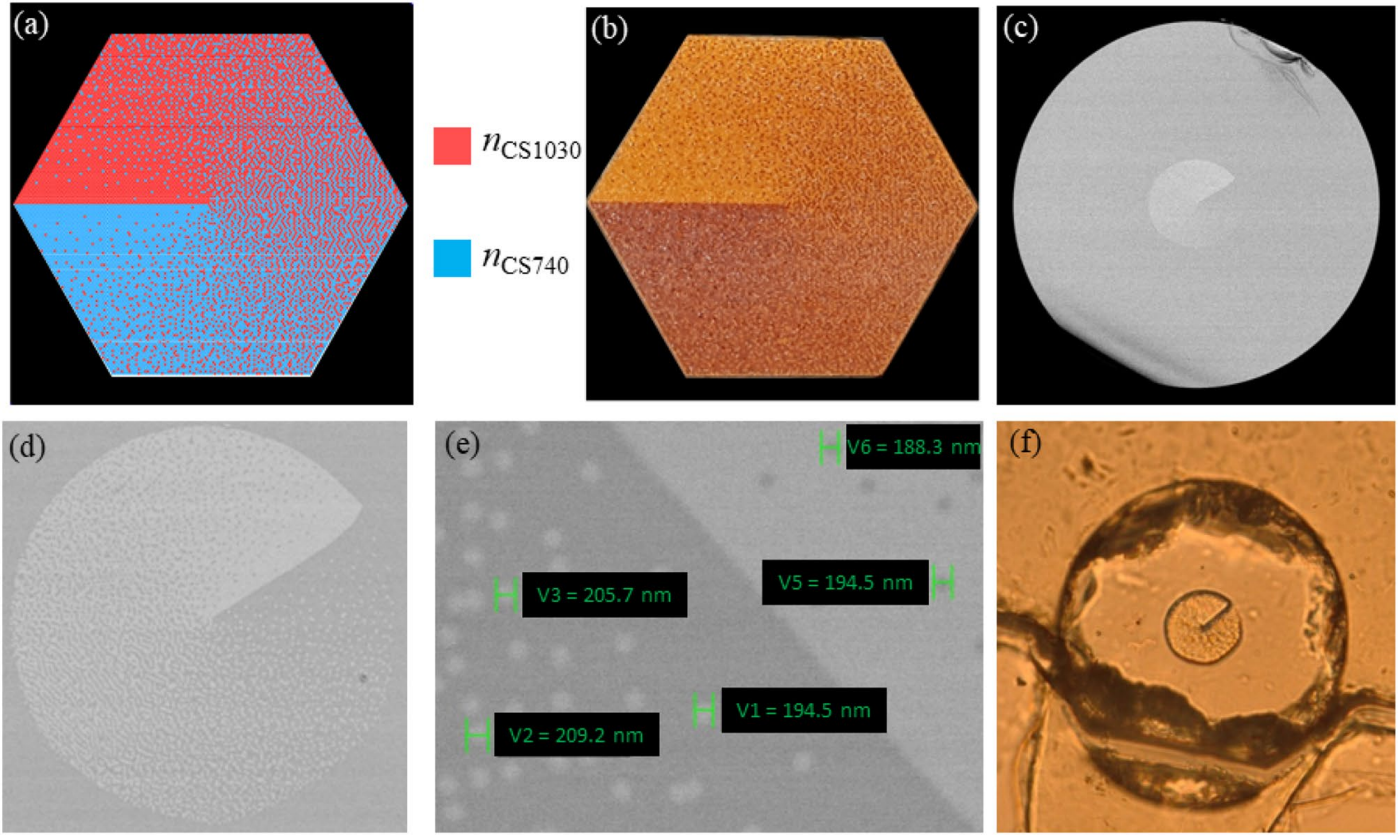
The design of binary-nanostructured vortex component (**a**) and its corresponding stacked preform (**b**); SEM images of the fabricated BnVPM sample (**c**) and its view under the microscope (**f**); the magnification of vortex structure (**d**) and its magnified part showing the size of individual nano-rods (**e**).

Our binary structure component as shown in Fig. 8a–f is composed of 17,557 rods in total, including 8907 CS1030-glass rods and 8650 CS740-glass rods. Its vortex structure diameter is 29 μm, with 153 nano-rods on the structure diagonal. That means the individual rod has a size of around 200 nm which is well below the threshold value for the infrared regimes for application of effective medium theory. This is confirmed by a scanning electron microscopy (SEM) image presented in Fig. 8d,e. Our fabricated BnVPM with the thickness of* d* = 19 µm (Fig. 8f) was designed to work with the most popular near infrared sources.

## Data Availability

The datasets generated during and/or analyzed during the current study are available from the corresponding author upon reasonable request.
